# Mechanical digit sensory stimulation: a randomized control trial on neurological and motor recovery in acute stroke

**DOI:** 10.3389/fnins.2023.1134904

**Published:** 2023-05-23

**Authors:** Shuting Zhang, Yang Yu, Panpan Xu, Xianshan Shen, Chuanqin Fang, Xiaosan Wu, Ping Qu, Tingting Wu, Qing Mei Wang, Xun Luo, Yongfeng Hong

**Affiliations:** ^1^Department of Rehabilitation Medicine, The Second Affiliated Hospital of Anhui Medical University, Hefei, Anhui Province, China; ^2^Department of Neurology, The Second Hospital of Anhui Medical University, Hefei, Anhui Province, China; ^3^Key Laboratory of Oral Disease Research of Anhui Province, Stomatologic Hospital and College, Anhui Medical University, Hefei, Anhui Province, China; ^4^Stroke Biological Recovery Laboratory, Spaulding Rehabilitation Hospital, Harvard Medical School, Boston, MA, United States; ^5^School of Medicine, Shenzhen University, Shenzhen, Guangdong Province, China

**Keywords:** acute ischemic stroke, mechanical digit sensory stimulation, IL-17A, TNF-α, VEGF-A

## Abstract

**Background:**

Mechanical digit sensory stimulation (MDSS) is a novel therapy designed to accelerate the recovery of upper limb (including hand) function in patients with hemiplegia following a stroke. The primary goal of this study was to investigate the effect of MDSS on patients with acute ischemic stroke (AIS).

**Methods:**

Sixty-one inpatients with AIS were randomly divided into conventional rehabilitation group (RG) and stimulation group (SG), and the latter group received MDSS therapy. A healthy group consisting of 30 healthy adults was also included. The interleukin-17A (IL-17A), vascular endothelial growth factor A (VEGF-A), and tumor necrosis factor-alpha (TNF-α) plasma levels were measured in all subjects. The neurological and motor functions of patients were evaluated using the National Institutes of Health Stroke Scale (NIHSS), Mini-Mental State Examination (MMSE), Fugel-Meyer Assessment (FMA), and Modified Barthel Index (MBI).

**Results:**

After 12 days of intervention, the IL-17A, TNF-α, and NIHSS levels were significantly decreased, while the VEGF-A, MMSE, FMA, and MBI levels were significantly increased in both disease groups. No significant difference was observed between both disease groups after intervention. The levels of IL-17A and TNF-α were positively correlated with NIHSS but negatively correlated with MMSE, FMA, and MBI. The VEGF-A levels were negatively correlated with NIHSS but positively correlated with MMSE, FMA, and MBI.

**Conclusion:**

Both MDSS and conventional rehabilitation significantly reduce the production of IL-17A and TNF-α, increase the VEGF-A levels, and effectively improve cognition and motor function of hemiplegic patients with AIS, and the effects of MDSS and conventional rehabilitation are comparable.

## 1. Introduction

Acute ischemic stroke (AIS) is a medical emergency with high morbidity, mortality, and disability rates worldwide, resulting in various degrees of cognitive and motor dysfunction, even after rehabilitation treatment (Tsivgoulis et al., 2016). According to statistics from the World Health Organization, the incidences of cognitive impairment and motor dysfunction of the upper limbs caused by stroke are around 37 and 60% in the sixth month of the disease, respectively (Arba et al., 2018; Dawson et al., 2021).

Mechanical digit sensory stimulation (MDSS), which primarily stimulates pressure sensation, is a novel therapy designed by our team to accelerate the recovery of the upper limb (including hand) function in patients with hemiplegia after stroke. We have developed a digit sensory stimulator (DSS) with a screen that can display the intensity of stimulation in real time. Our previous clinical observation of more than 1 year suggests that DSS is suitable for clinical application and can promote the cognitive and limb motor functions of patients after stroke.

Chandrasekaran et al. found that the sulcal stimulation in the primary somatosensory cortex (S1) could induce sensory perception located in the fingertip better than the gyral stimulation, and the perception caused by the sulcal stimulation was often highly concentrated in a single segment of the finger, especially the distal phalanx including the fingertip (Chandrasekaran et al., 2021). Kattenstroth et al. pointed out that compared with any other part of the body, fingertips and toetips are more sensitive sites with a higher density of nerve endings and somatosensory receptors, and these sites are the most innervated, and stimulation of these two sites can induce the excitement of specific targeted somatosensory cortical areas (Kattenstroth et al., 2018). Appropriate sensory stimulation of the fingers/toes in some patients with central nervous system injury can induce more cerebral cortical excitation and limb movements (Dobkin, 2003; Kattenstroth et al., 2018; Ayoobi et al., 2021). Therefore, the sites of sensory stimulation were concentrated on the hemiplegic finger/toenail beds to achieve good results in this study. In addition, Kattenstroth et al. reported that electrical stimulation of the affected fingertip of patients with subacute stroke for 2 weeks significantly improved their upper limbs’ sensory and motor function (Kattenstroth et al., 2018). However, the specific effects of MDSS on hemiplegic patients with AIS remain unclear.

Previous studies have shown that inflammatory response significantly contributes to ischemic brain injury (Bustamante et al., 2016; Nakamura and Shichita, 2019). Interleukin-17A (IL-17A), an essential immune cytokine, aggravates cerebral ischemia in the acute stage of ischemic stroke by upregulating inflammatory mediators and chemokines (Liu et al., 2019). Tumor necrosis factor-alpha (TNF-α) is a pro-inflammatory cytokine involved in neuroinflammation and neuronal damage induced by cerebral ischemia (Lambertsen et al., 2019). It also promotes the release of neurotoxic substances and aggravates ischemic injury (Lambertsen et al., 2012; Bokhari et al., 2014; Chen et al., 2019). Vascular endothelial growth factor A (VEGF-A) is a crucial cytokine responsible for the regulation of angiogenesis, which promotes neurogenesis and nerve function recovery after ischemic stroke (Lucitti et al., 2012; Ma et al., 2012). Taken together, IL-17A, TNF-α, and VEGF-A are closely related to ischemic stroke occurrence and development (Kawabori and Yenari, 2015).

Somatosensory stimulation of the brain of some patients with central nervous system injury through their fingers may affect the metabolism and functional recovery of the brain (Dobkin, 2003; Ayoobi et al., 2021). Previous studies have revealed that ischemic stroke temporarily but significantly increased IL-17A, TNF-α, and VEGF-A levels (Arango-Dávila et al., 2015; Liu et al., 2019; Moon et al., 2021; Xu et al., 2022). Therefore, the purpose of this study was to investigate the effects of MDSS on the plasma levels of cytokines and the cognitive and motor functions of hemiplegic patients with AIS and to explore its noninferiority in terms of rehabilitation outcomes when compared with the conventional therapy.

## 2. Methods

### 2.1. Participants

In this single-center, randomized study, the hemiplegic patients with AIS admitted to the Department of Neurology in the Second Hospital of Anhui Medical University between December 2020 and September 2022 were recruited. A healthy group of some age-and gender-matched healthy adults were also enrolled. This study was approved by the Medical Ethics Committee of the Second Hospital of Anhui Medical University [Approval No. YX2020-030(F1)] and performed in accordance with the Declaration of Helsinki. Written informed consent was obtained from each participant before enrollment.

### 2.2. Inclusion and exclusion criteria

Healthy subjects were recruited from the workers, interns, and ward attendants of Department of Rehabilitation Medicine in the Second Hospital of Anhui Medical University. The inclusion criteria of healthy subjects were as follows: (a) aged between 18 and 80 years; (b) clear consciousness and normal cognition; (c) no history of heart disease, stroke, rheumatic disease, acute gout, tumor, tuberculosis, infection (including viral and bacterial), new fracture, etc. (d) willing to sign informed consent. All healthy subjects were asked to ensure adequate sleep and normal mental state before the experiment, and to avoid smoking and intake of irritating foods, such as alcohol, tea, and coffee.

Patients who met the following criteria were qualified for inclusion: (a) aged between 18 and 80 years; (b) diagnosed with partial anterior circulation infarct (PACI) for the first time with the diagnosis confirmed by magnetic resonance imaging or computerized tomography scan (Novotny et al., 2021); (c) hospitalized within 48 h of ischemic stroke onset with stable vital signs (systolic blood pressure, 90–160 mmHg; diastolic blood pressure, 60–100 mmHg; oxygen saturation, > 92%; resting heart rate, 60–110 beats/min; and body temperature, ≤38°C); (d) clear consciousness and willing and able to cooperate with the assessments and treatments; (e) the strength of all muscles of the upper limb, including shoulder, elbow, wrist, and hand, and the lower limb, including hip, knee, ankle, and foot, on the hemiplegic side was ≤ grade 3 in the manual muscle testing (Ciesla et al., 2011), while that on the unaffected side showed no dysfunction; and (f) self-identified as right-handed and confirmed by his/her family member.

The exclusion criteria for patients were as follows: (a) two or more strokes before the study; (b) other types of AIS other than PACI; (c) evidence of continued deterioration of the condition with unstable vital signs; (d) complications with heart, lung, liver, or renal insufficiency or a malignant tumor; (e) pregnant or lactating; (f) treated with antibiotics for an infection in any site, such as the lung or the urinary tract; and (g) currently participating in other clinical trials.

### 2.3. Mechanical digit sensory stimulation

**Figure 1:**
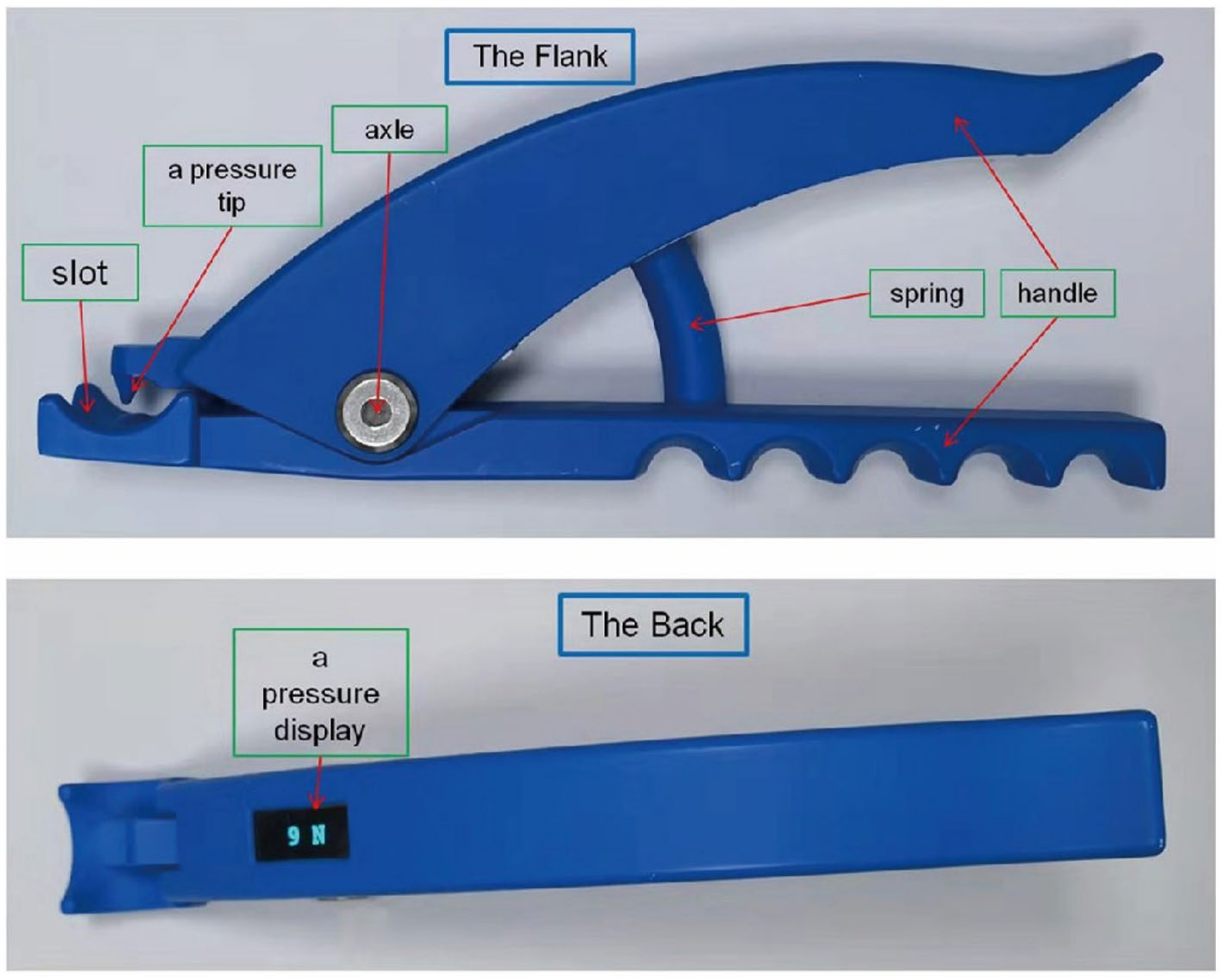
Digit sensory stimulator.

**Figure 2:**
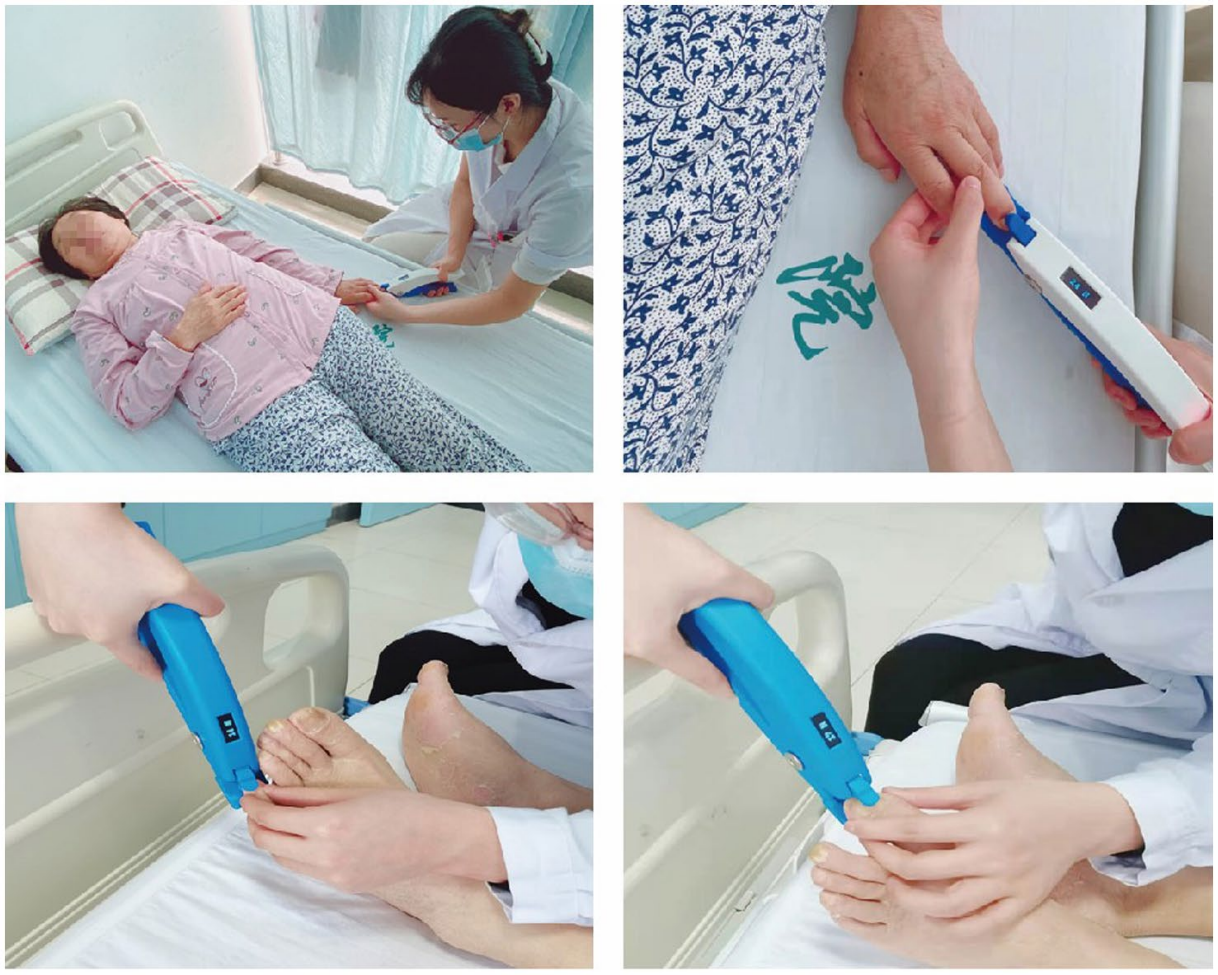
Implementation of mechanical digit sensory stimulation.

The DSS device consists of a handle, a spring, an axle, a pressure tip, a card slot, and a screen displaying the pressure (Figure 1). During the MDSS therapy, the operator held the device’s handle and placed the pressure tip at the hemiplegic side’s fingernail/toenail bed root. Then, the operator relaxed the handle relatively quickly, so the pressure tip could rapidly stimulate the fingernail/toenail bed. If the stimulation intensity was acceptable to the patient, the operator gripped the device’s handle to stop stimulation as soon as the extension of the stimulated fingers/toes or the retraction of the stimulated upper/lower limbs was observed. However, if the stimulation intensity exceeded the acceptable range, the operator stopped stimulation immediately, even if finger/toe extension or limb retraction was not observed. The screen on the reverse of the device displayed real-time stimulation intensity in Newtons (N), as shown in Figure 2.

### 2.4. Grouping and treatment

After screening for eligibility by a medical doctor, hemiplegic patients with AIS were randomly assigned to two groups: the stimulation group (SG) that received MDSS therapy with the DSS (Figures 1, 2) and the rehabilitation group (RG) that received conventional rehabilitation without the MDSS.

All patients received comprehensive neurological treatment according to the Guidelines for the Early Management of Patients with Acute Ischemic Stroke (Powers et al., 2019). Patients in the RG received conventional rehabilitation treatment, including body massage, breathing exercises, active movement of the unaffected limb, active/assisted/passive movement of the hemiplegic limbs according to the patient’s condition, and neuromuscular electrical stimulation of the upper and lower limbs on the hemiplegic side. In the RG, all treatments were administered once a day, with daily treatment lasting 50–70 min.

Body massage was applied only to the upper and lower limbs of the hemiplegic side. Since all patients were in the acute phase of cerebral infarction, the muscle tone of the upper and lower limbs on the hemiplegia side was low, so we used rapid manual massage with moderate force for the flexor and extensor muscles of the hemiplegic limb, once a day for 10 min. The breathing exercises were carried out by inhaling deep through the nose, pouting and blowing hard through the mouth, mainly to train abdominal breathing. Each set of breathing exercises consisted of 5 repetitions, and 3–5 sets were performed consecutively, with a 30-second break between two sets, a total of 5–10 min, and 3–5 sets per day. Movement patterns of the unaffected and affected limbs were mainly forward flexion and abduction of the upper limb and straight leg raising, overall flexion and extension of the lower limb in the supine position. During the movement of the unaffected limb, the unaffected hand held a bottle of mineral water or a proper weight sandbag was tied around the unaffected wrist/ankle. Active/assisted/passive movements were selected according to the muscle strength of the upper and lower limbs on the hemiplegic side. Each movement of the limb was performed 10 times in a set, with an interval of 30 s between two sets, a total time of 20–30 min per day. We used low-frequency electrical stimulation of the upper and lower limbs on the hemiplegic side, and the specific sites of electrical stimulation were selected according to the residual muscle strength. The biceps brachii and quadriceps femoris were first selected as targets for electrical stimulation. However, if the muscle strengths of the biceps brachii and quadriceps femoris were ≥ grade 2 (manual muscle testing, MMT), their antagonist muscles were electrically stimulated, and the upper and lower limbs were electrically stimulated at the same time, for 15–20 min once daily.

Instead of conventional rehabilitation, patients in the SG received MDSS therapy. The same investigator performed the therapy for all patients in the group. With the patient in a supine position, the investigator stimulated the nail bed root of all five fingers and toes separately on the hemiplegic side using the DSS, from the little finger/toe to the thumb/big toe. The stimulation pressure was between 30 and 50 N, enough to cause active extension of the fingers/toes or retraction of the upper/lower limbs on the hemiplegic side and was tolerable for the patient. The stimulation duration for each finger/toe was 2–3 s, and the interval between two adjacent stimulations was 5–10 s. The total stimulation time of five fingers and five toes was 70–120 s. Based on ethical grounds and in view of the short time required for each stimulation, patients in the SG were treated three times daily, once in the morning, once at noon, and once in the evening to guarantee that all patients have access to effective treatment.

**Figure 3:**
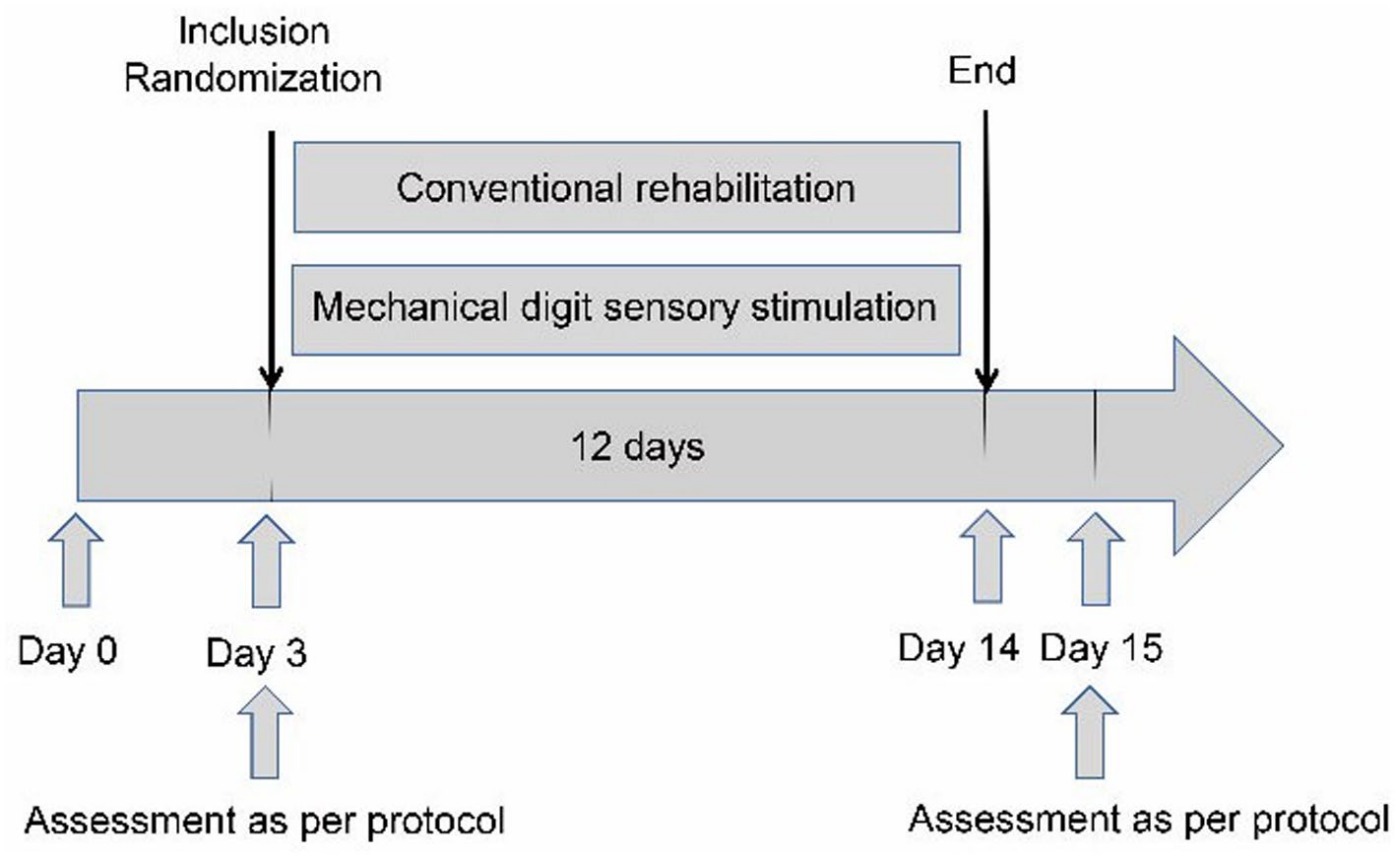
Flow chart.

The interventions (conventional rehabilitation in the RG and MDSS therapy in the SG) started from the third day after disease onset and continued for 12 days (until the 14th day after disease onset). The timeline of the interventions is shown in Figure 3.

### 2.5. Safety and acceptability evaluation

All patients were observed for discomfort, seizure, recurrent cerebral hemorrhage or infarction, pulmonary embolism, myocardial infarction, and death during the study. After the first and last stimulation, patients in the SG were asked to self-assess the degree of pain caused by MDSS using the visual analog scale (VAS): 0, no pain; 1–3, mild pain; 4–6, marked pain; and 7–10, intense pain (Sung and Wu, 2018). The acceptance was evaluated by a subjective 4-item questionnaire after 12 days of intervention: (Q1) “Is the therapy with the MDSS motivating?” (0 no, 1 yes), (Q2) “Would you recommend the MDSS to other subjects with stroke?” (0 no, 1 yes), (Q3) “Has the MDSS therapy led to concrete improvements?” (0 no, 1 yes), (Q4) “How comfortable was the therapy with the MDSS for you?” (0 uncomfortable, 10 very comfortable; Ranzani et al., 2020).

### 2.6. Measurement of plasma cytokines levels

Fasting venous blood (3 ml) was collected in the morning from each subject in the disease groups before intervention (the third day after disease onset) and after 12 consecutive days of intervention (the 15th day after disease onset). The same amount of fasting venous blood was collected from each subject in the healthy group immediately after enrollment. Blood samples were centrifuged at 3,000 rpm for 15 min. Then, the plasma was isolated using a pipette and stored at −80°C until analysis (Tang et al., 2017). The IL-17A, TNF-α, and VEGF-A plasma levels were determined using enzyme-linked immunosorbent assay kits (Shanghai Jianglai Industrial Co., Ltd., Shanghai, China) according to the manufacturer’s instructions (Aydin, 2015; Eble, 2018).

### 2.7. Neurological and motor function assessment

The neurological and motor functions of each patient were evaluated using multiple assessment scales before and after intervention. The National Institutes of Health Stroke Scale (NIHSS) was used to evaluate neurological deficits. The total score ranges between 0 and 42, with higher scores indicating more severe nerve damage. The NIHSS is scored as follows: 0–1, normal; 2–4, mild impairment; 5–15, moderate impairment; 16–20, moderate to severe impairment; and ≥21, severe impairment (Lyden, 2017). The Mini-Mental State Examination (MMSE) was used to evaluate cognitive function. It consists of 19 items with a total of 30 points. Higher scores indicate better cognitive function. A score of ≤26 indicates cognitive impairment, while a score of 27–30 is considered within the normal range (Körver et al., 2019). The Fugel-Meyer Assessment (FMA) was used to evaluate the motor function of the upper and lower limbs and has a total score of 100. Higher scores indicate better limb motor function. A score of 0–50 suggests severe limb paralysis, a score of 51–84 indicates significant limb paralysis, a score of 85–95 denotes moderate limb paralysis, and a score of 96–100 represents mild limb paralysis (Fugl-Meyer et al., 1975). The Modified Barthel Index (MBI) was used to assess the ability to complete activities of daily living with a total score of 100. The higher the score, the higher the ability to complete daily living activities. The MBI is scored as follows: 0–20, severe functional impairment; 25–45, serious functional impairment; 50–70, moderate functional impairment; 75–95, mild functional impairment; and 95–100, normal (Taghizadeh et al., 2020). The primary outcome of the study, which was tested for equivalence, was the change in motor impairment at the end of treatment relative to before intervention, assessed by the FMA. The FMA scale was used as the primary outcome measure due to its widespread use in sensorimotor rehabilitation assessment (Ranzani et al., 2020).

### 2.8. Statistical analysis

The sample size was estimated to achieve a power (1 − β) of 0.8 to detect a 4.66-point difference in FMA (Metzger et al., 2014), with a 2-sided significance level of 0.05. A total of 27 patients in each group were required. Allowing for a 10% loss, 30 patients in each group should be recruited, for a total of 60 patients. The sample size was determined using the G*Power software (version 3.1.9.7). Data were analyzed using the SPSS 25.0 software. The chi-square test compared categorical variables. All quantitative variables were tested for normal distribution using the Shapiro–Wilk test. Normally distributed data were expressed as mean ± standard deviation (SD). The independent sample t-test was used to compare quantitative variables between the two disease groups; The paired sample t-test was used to compare the data before and after intervention in each group. Pearson correlation analysis was used to determine the correlations of plasma levels of IL-17A, TNF-α, and VEGF-A with the score of each assessment. A value of *p* < 0.05 indicated statistical significance. Equivalence analysis was used to investigate whether the two disease groups showed an equivalent change in terms of the primary outcome measure (Walker and Nowacki, 2011). Equivalence was established if the difference in the FMA score between the two disease groups lay within an equivalence boundary of ±5.2 points, which was reported to be the minimal detectable/clinically important difference for the FMA (Wagner et al., 2008; Ranzani et al., 2020).

### 2.9. Data availability statement

The data associated with the paper are not publicly available but are available from the corresponding author upon reasonable request.

## 3. Results

**Figure 4:**
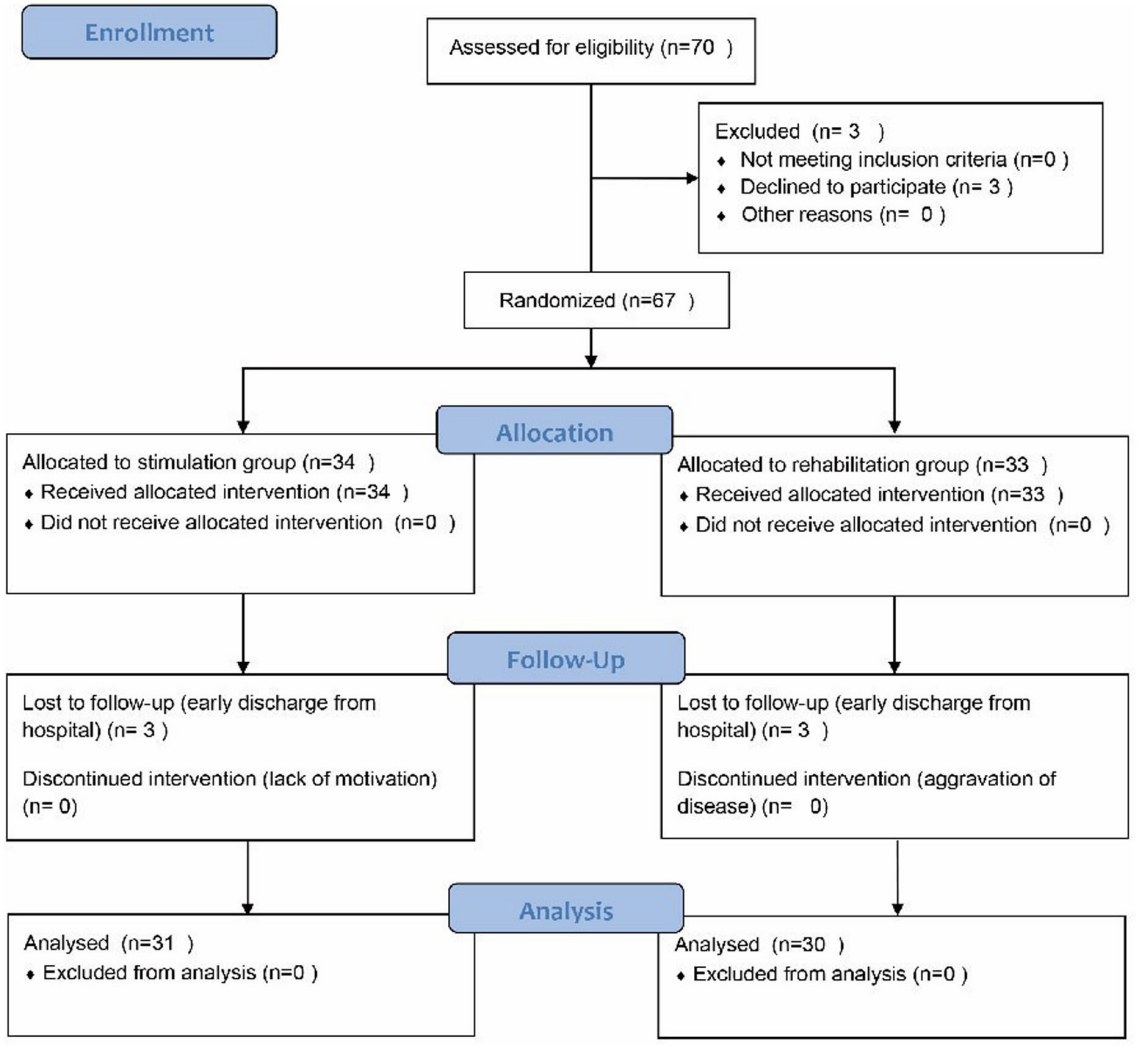
Trial profile describing the participants population.

Sixty-seven subjects with AIS were eligible and consented to participate in the study, 33 of whom were randomly assigned to the RG and 34 to the SG. Six (3 in the RG and 3 in the SG) subjects did not complete the intervention protocol due to early discharge from hospital and dropped out halfway through the study. Only 61 subjects (30 in the RG and 31 in the SG) received corresponding interventions and assessments (Figure 4). No adverse event related to the intervention was observed during the study. A healthy group consisting of 30 age-and gender-matched healthy adults were also enrolled.

### 3.1. Baseline characteristics

The demographics and clinical characteristics of different groups at baseline are summarized in Table 1. Participants were aged between 26 and 80 years. No statistically significant differences were found in gender, age, and history of smoking, drinking, diabetes, obesity, hypertension, intravenous thrombolysis or arterial embolectomy among the three groups (*p* > 0.05).

**Table 1:** Baseline characteristics of the randomized study participants.

Characteristics	Rehabilitation group (*n* = 30)	Stimulation group (*n* = 31)	Healthy group (*n* = 30)	*χ*^2^/F	*p*
Age (years) mean (SD)	68.73 (11.08)	63.68 (12.60)	65.03 (6.19)	1.938	0.150
Sex (Male/Female)	1614	22/9	16/14	2.634	0.268
Smoking history (%)	13 (43.33%)	17 (54.84%)	10 (33.33%)	2.869	0.238
Drinking history (%)	11 (36.67%)	12 (38.71)	13 (43.33%)	0.293	0.864
Diabetes (%)	9 (30%)	11 (35.49%)	12 (40%)	0.660	0.719
Obesity (%)	4 (13.33%)	10 (32.26%)	9 (30%)	3.420	0.181
Hypertension (%)	25 (83.33%)	25 (80.65%)	23 (76.67)	0.426	0.808
Intravenous thrombolysis or arterial embolectomy (%)	6 (20%)	9 (29.03%)	-	0.671	0.554

### 3.2. Plasma levels of IL-17A, TNF-α, and VEGF-A

The IL-17A, TNF-α, and VEGF-A plasma levels in the RG and SG before intervention were significantly higher than those in the healthy group (all *p* < 0.01), but no significant difference was observed between the two disease groups (*p* > 0.05). The IL-17A and TNF-α levels in both disease groups after intervention were significantly decreased, while those of VEGF-A were significantly increased compared with those before intervention (all *p* < 0.01). However, no significant difference in cytokine levels was observed between the RG and SG after intervention (*p* > 0.05). Compared with those in the healthy group, the IL-17A and VEGF-A levels in the RG and SG after intervention were significantly increased (*p* < 0.01), while the TNF-α levels were not significantly different (*p* > 0.05). As shown in Table 2.

**Table 2:** Plasma levels of IL-17A, TNF-α, and VEGF-A in different groups (pg/ml).

Cytokine	Healthy Group mean (SD)	Disease groups before intervention mean (SD)			Disease groups after intervention mean (SD)		
		Rehabilitation group	Stimulation group	*p*	Rehabilitation group	Stimulation group	*p*
IL-17A	14.45 (1.63)	24.76 (6.33)^#^	23.44 (6.14)^#^	0.322	18.53 (6.99)^*#^	17.46 (6.52)^*#^	0.538
TNF-α	31.83 (6.37)	50.78 (7.37)^#^	46.90 (9.37)^#^	0.056	36.38 (11.35)^*^	31.00 (10.84)^*^	0.063
VEGF-A	123.30 (9.55)	142.19 (23.96)^#^	138.40 (28.08)^#^	0.542	189.95 (37.84)^*#^	198.83 (29.85)^*#^	0.312

Intra-group comparison before and after intervention, ^*^p < 0.01; compared with Healthy Group, ^#^*p* < 0.01.

### 3.3. Results of assessment scales

**Figure 5:**
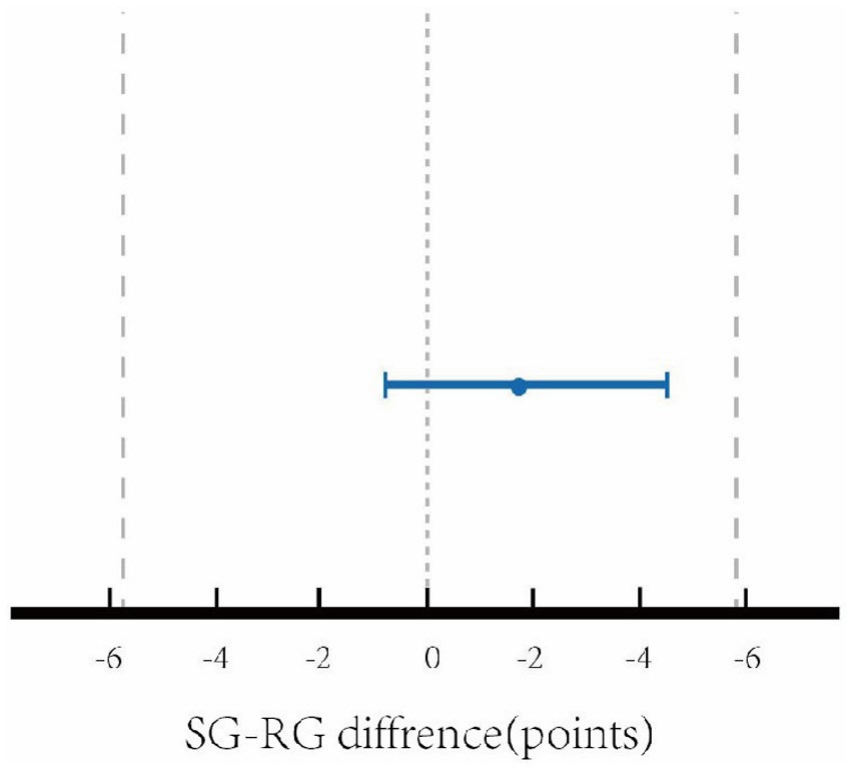
Equivalence analysis between stimulation group and rehabilitation group with reference to the FMA change.

The NIHSS, MMSE, FMA, and MBI scores were not significantly different between the RG and SG before intervention (*p* > 0.05). The NIHSS scores were significantly decreased, while the MMSE, FMA, and MBI scores were significantly increased in the two disease groups after intervention (all *p* < 0.01). However, no significant difference in the assessment scales’ results after intervention was observed between the two disease groups (*p* > 0.05). As shown in Table 3. According to the equivalence analysis (Figure 5), the change in the FMA score in the SG could be considered as non-inferior to that in the RG. The 90% confidence interval lay within the equivalence boundaries in favor of the MDSS therapy at the end of the study. Before and after intervention, subjects in the SG improved on average by 17.90 FMA points, while those in the RG showed an average increase of 17.47 FMA points. In both disease groups, these changes were above the minimal detectable/clinically important difference (Shelton et al., 2001; Wagner et al., 2008).

**Table 3:** Results of NIHSS, MMSE, FMA, and MBI before and after intervention.

Scale	Before intervention mean (SD)			After intervention mean (SD)		
	Rehabilitation group	Stimulation group	*p*	Rehabilitation group	Stimulation group	*p*
NIHSS	8.87 (2.79)	8.26 (3.67)	0.470	4.53 (1.68)^*^	4.52 (2.86)^*^	0.977
MMSE	20.40 (3.25)	21.74 (3.32)	0.116	23.27 (2.57)^*^	24.52 (2.84)^*^	0.077
FMA	54.27 (11.93)	55.61 (10.72)	0.645	71.73 (10.24)^*^	73.52 (11.16)^*^	0.519
MBI	36.50 (13.01)	37.90 (11.75)	0.660	62.50 (9.54)^*^	66.61 (13.75)^*^	0.181

Intra-group comparison before and after intervention.

* *p* < 0.01.

### 3.4. Correlation analysis

The levels of IL-17A and TNF-α before and after intervention were positively correlated with the NIHSS scores, but negatively correlated with the MMSE, FMA, and MBI scores (*p* < 0.01); The VEGF-A levels before and after intervention were negatively correlated with the NIHSS scores but positively correlated with the MMSE, FMA, and MBI scores (*p* < 0.01). As shown in Table 4.

**Table 4:** Correlations of cytokine levels with the results of each assessment scale.

Cytokine	NIHSS (r/*p*)	MMSE (r/*p*)	FMA (r/*p*)	MBI (r/*p*)
IL-17A	0.2664/0.003	−0.3628/0.0001	−0.2810/0.0017	−0.3724/0.0001
TNF-α	0.3057/0.0006	−0.4098/0.0001	−0.3755/0.0001	−0.4890/0.0001
VEGF-A	−0.4137/0.0001	0.3784/0.0001	0.4848/0.0001	0.5231/0.0001

### 3.5. Acceptance of MDSS therapy

All patients in the SG had a VAS score of ≤6, and the mean VAS score of this cohort was 1.25 ± 1.06, suggesting that the pain caused by MDSS was mild. Of 31 participants who answered the questionnaire in the SG, 90.32% found the MDSS therapy motivating (Q1), 83.87% would recommend the MDSS therapy program to other persons with stroke (Q2), and 87.10% experienced concrete improvements in their health status at the end of the therapy program (Q3). Participants rated the MDSS treatment as comfortable with a score of 7.81 ± 1.25 out of 10 (Q4).

## 4. Discussion

This study shows that MDSS is a safe and effective therapy for patients with AIS. Treatment with the DSS decreased inflammatory factors (i.e., IL-17A and TNF-α), increased nerve regeneration factor VEGF-A levels, alleviated neurological impairment, and restored cognitive and limb motor functions. The result of the equivalence analysis comparing the evolution in the FMA demonstrated that the motor recovery in the stimulation group is non-inferior with respect to the control group. The correlation analysis revealed that plasma cytokine levels were correlated with the results of clinical assessment scales.

Previous studies have shown that a time window of neuroplasticity exists in the early post-stroke period, during which the brain’s dynamic response to rehabilitation treatment is sensitive and robust (Coleman et al., 2017). There is no consensus on the optimal time to start rehabilitation after stroke. However, increasing evidence has demonstrated that intensive rehabilitation treatment within 24 h after stroke might harm patients, while treatment within the first 2 weeks after stroke will alleviate functional impairment (Li et al., 2017). Early rehabilitation, which starts between 24 and 72 h after stroke, has been shown to reduce the levels of inflammatory cytokines, protect the blood–brain barrier, inhibit apoptosis, promote neurogenesis, and upregulate brain-derived neurotrophic factor levels (Kim et al., 2005; Zhang et al., 2013). Li et al. found that exercise within 6–24 h after stroke increased the plasma levels of inflammatory cytokines, while the same exercise starting from the third day after stroke reduced the production of these cytokines (Li et al., 2017). Consistent with this, Tang et al. treated patients with AIS with di-3-n-butylphthalide from the third to the 14th day after disease onset and observed increased serum levels of VEGF and basic fibroblast growth factor after treatment (Tang et al., 2017). Therefore, in this study, all interventions started from the third day after disease onset.

Neurological changes after stroke are significantly associated with pro-inflammatory and anti-inflammatory cytokines (Yan et al., 2015). The imbalance and balance between these cytokines play a vital role in the processes of nerve injury and repair after stroke (Vila et al., 2000). Lin et al. found that IL-17A is released by γδT cells, peaks on day three after stroke, and aggravates ischemic brain injury (Lin et al., 2016). The TNF-α levels are significantly increased after AIS. This increase may lead to the activation of neutrophils, which enhances white blood cell phagocytosis, promotes the secretion of inflammatory cytokines, and eventually increases vascular permeability and aggravates edema (Camelo et al., 2001; Shi et al., 2017). In addition, VEGF-A stimulates the proliferation and migration of vascular endothelial cells, accelerates new blood vessel formation, and constructs a collateral circulation network that can save the ischemic penumbra (Lucitti et al., 2012). The neuroprotective effect of VEGF-A has also been observed in a rat model of middle cerebral artery embolization (Liang et al., 2020).

In this study, we found that the IL-17A, TNF-α, and VEGF-A plasma levels of patients with AIS on day three after disease onset were significantly higher than those of the healthy group. Both the IL-17A and TNF-α levels were significantly decreased, while the VEGF-A levels were significantly increased in the RG and SG after intervention. These findings were consistent with the studies by Lin et al. (2016), Tang et al. (2017), and Chen and Zhao (2018). Moreover, compared to the results obtained before intervention, the NIHSS scores were significantly decreased, while the scores of MMSE, FMA, and MBI were significantly increased in both groups of AIS patients on the 15th day after disease onset. Similarly, the study by Kattenstroth et al. showed that the rehabilitation including repetitive sensory stimulation was more effective than standard therapy alone in sensorimotor recovery (Kattenstroth et al., 2018). According to previous studies and our experimental results, the primary measures (the FMA scores) of the patient’s function in the rehabilitation and stimulation groups exceeded the minimal clinically important difference (+5.2 points) after corresponding treatment, indicating not only statistical differences, but also clinical significance (Hsieh et al., 2007; Cervera et al., 2018). The correlation analysis showed that the IL-17A and TNF-α levels were negatively correlated with the MMSE, FMA, and MBI scores (*p* < 0.01), and the VEGF-A level was positively correlated with the MMSE, FMA, and MBI scores (*p* < 0.01). These results suggest that IL-17A and TNF-α may inhibit cognitive and motor recovery, while VEGF-A may alleviate brain injury and accelerate brain function recovery at an early stage following ischemic stroke, which is in line with the results from previous studies (Rreed et al., 2020; Zuo et al., 2022).

Data on the 15th day after disease onset showed that the NIHSS score was significantly decreased, while the MMSE, FMA, and MBI scores were significantly increased in both disease groups after intervention, which was consistent with the findings by Kattenstroth et al. (2018). In addition, the rehabilitation and stimulation groups showed significantly lower levels of IL-17A and TNF-α, which tended to be at normal range, and higher levels of VEGF-A, which deviated even more from normal after intervention. These results suggest that both conventional rehabilitation and MDSS therapy were effective in inhibiting the secretion of pro-inflammatory cytokines (i.e., IL-17A and TNF-α) and promoting the production of nerve regeneration factor VEGF-A, thereby reducing neurological impairment and accelerating cognitive and limb motor function recovery (Zhang et al., 2021; Cha et al., 2022). There was no significant difference in the IL-17A, TNF-α, and VEGF-A levels, or the NIHSS, MMSE, FMA, and MBI results between the rehabilitation and stimulation groups after intervention. Furthermore, the equivalent analysis of FMA changes showed that the motor function recovery of the stimulation group was non-inferior to the rehabilitation group. MDSS is essentially a pressure stimulation that can be considered as a variation of the Rood technique targeting the fingers and toes. The Rood technique is a multi-sensory stimulation therapy developed by Margaret Rood (Nielsen et al., 1986). It emphasizes the use of touch, squeeze, pull, vibration, percussion, and friction to produce different sensory stimulations, and to induce active muscle contraction while inhibiting antagonistic muscle contraction. Meanwhile, the Rood technique can input a variety of sensations into the central nervous system to improve the activity level of central neurons, thereby causing motor, cognitive, and other reactions, and promoting the recovery of neurological function (Bordoloi and Deka, 2019; Bordoloi and Deka, 2020; Chaturvedi and Kalani, 2023). In this study, DSS device was used to induce active retraction movements of the upper and lower limbs in patients with AIS by targeting the roots of the hemiplegic finger/toennail beds to produce tactile pressure sensation, mild pain sensation, and proprioception stimulation, which meet the definition of the Rood technique. Compared to stimulation on other parts of the body, stimulation of the fingertips and toetips, which are the most innervated regions, allows more specific targeting in the somatosensory cortical area (Kattenstroth et al., 2018). MDSS can achieve a good effect because the nerve endings and somatosensory receptors in fingers/toes are remarkably rich and sensitive. Appropriate mechanical stimulation of the fingers/toes on the hemiplegic side of patients with AIS can induce stronger excitement of the cerebral cortex and result in more limb movements, this can lead to a greater increase neuroplasticity and more significantly improvements in cognitive and motor functions compared to stimulation of other body part (Dobkin, 2003; Kattenstroth et al., 2018; Ayoobi et al., 2021). Conventional rehabilitation often requires expensive equipment (e.g., neuromuscular electrical stimulation), which costs a few hundred to tens of thousands of Chinese Yuan and takes 20–30 min per session. The DSS is a less-costly, safe, and user-friendly device and MDSS therapy only takes 70–120 s per session. In addition, our results showed that the VAS scores of all patients after MDSS were ≤6, and the mean VAS score of this cohort was 1.25 ± 1.06, indicating that the pain caused by MDSS was within the acceptable range for patients. More importantly, caregivers and family members can implement MDSS using the DSS after simple training, allowing MDSS application both in the ward and at home.

The present study has some limitations. Firstly, the long-term effects of MDSS remain unknown due to the short observation period. Secondly, the effects of MDSS on different subgroups were not explored because the sample size of this study was relatively small. Finally, the researchers were not blinded to grouping. To maintain experimental rigor, it would be necessary to establish a group that does not receive rehabilitation treatment; however, such a group would be considered unethical. Further studies with larger sample sizes, more extended observation periods, and inclusion of patients with unclear consciousness due to AIS need to be performed, and the experiment should be blinded.

## 5. Conclusion

In conclusion, this randomized, controlled trial demonstrates that IL-17A and TNF-α play an inflammatory role in the acute and subacute stages of stroke and aggravate brain injury, while VEGF-A exerts a neuroprotective effect. MDSS inhibits the expressions of IL-17A and TNF-α, induces VEGF-A secretion, decreases the NIHSS score, and increases the MMSE, FMA, and MBI scores in patients with AIS. MDSS and conventional rehabilitation therapy enhance the neurological and motor functions of hemiplegic patients with AIS after 12 days of treatment, and the effectiveness of the two methods is comparable. Compared with conventional rehabilitation, MDSS is simpler, less time-consuming, less costly, and more suitable for hospital, community and home use.

## Data availability statement

The original contributions presented in the study are included in the article/Supplementary material, further inquiries can be directed to the corresponding authors.

## Ethics statement

The studies involving human participants were reviewed and approved by the Medical Ethics Committee of the Second Hospital of Anhui Medical University [Approval No. YX2020-030 (F1)]. The patients/participants provided their written informed consent to participate in this study.

## Author contributions

SZ, YY and PX: patient data collection. SZ: writing the first draft of the manuscript. TW, QW, XL and YH: study design, data analysis, and revising the manuscript. XS and CF: drawing the figures. XW and PQ: implementing rehabilitation treatment. All authors contributed to the article and approved the submitted version.

## Funding

This study was supported by the Scientific Research Fund Project from Anhui Academy of Translational Medicine (No. 2021zhyx-C50) and the Shenzhen Science and Technology Innovation Program (No. JCYJ20180305163652073).

## Conflict of interest

The authors declare that the research was conducted in the absence of any commercial or financial relationships that could be construed as a potential conflict of interest.

## Publisher’s note

All claims expressed in this article are solely those of the authors and do not necessarily represent those of their affiliated organizations, or those of the publisher, the editors and the reviewers. Any product that may be evaluated in this article, or claim that may be made by its manufacturer, is not guaranteed or endorsed by the publisher.
